# Development of the Brazilian Portuguese version of the Achilles Tendon Total Rupture Score (ATRS BrP): a cross-cultural adaptation with reliability and construct validity evaluation

**DOI:** 10.1186/s13102-016-0034-0

**Published:** 2016-04-21

**Authors:** Roberto Zambelli, Rafael Z. Pinto, João Murilo Brandão Magalhães, Fernando Araujo Silva Lopes, Rodrigo Simões Castilho, Daniel Baumfeld, Thiago Ribeiro Teles dos Santos, Nicola Maffulli

**Affiliations:** Orthopedic Department, Hospital Mater Dei, Belo Horizonte, Minas Gerais Brazil; Pain Management Research Institute, University of Sydney at the Royal North Shore Hospital, Sydney, Australia; Departamento de Fisioterapia, Faculdade de Ciências e Tecnologia, Universidade Estadual Paulista (UNESP), Presidente Prudente, São Paulo Brazil; Orthopedic Department, Hospital Felício Rocho, Belo Horizonte, Minas Gerais Brazil; Department of Physical Therapy, School of Physical Education, Physical Therapy and Occupational Therapy, Universidade Federal de Minas Gerais (UFMG), Belo Horizonte, MG Brazil; Department of Musculoskeletal Disorders, University of Salerno School of Medicine and Surgery, Salerno, Italy; Centre for Sports and Exercise Medicine, Queen Mary University, Mile End Hospital, London, UK

**Keywords:** Achilles Tendon Total Rupture Score (ATRS), Complete rupture of the Achilles Tendon, Functional questionnaires, Cross-cultural adaptation

## Abstract

**Background:**

There is a need for a patient-relevant instrument to evaluate outcome after treatment in patients with a total Achilles tendon rupture. The purpose of this study was to undertake a cross-cultural adaptation of the Achilles Tendon Total Rupture Score (ATRS) into Brazilian Portuguese, determining the test-retest reliability and construct validity of the instrument.

**Methods:**

A five-step approach was used in the cross-cultural adaptation process: initial translation (two bilingual Brazilian translators), synthesis of translation, back-translation (two native English language translators), consensus version and evaluation (expert committee), and testing phase. A total of 46 patients were recruited to evaluate the test-retest reproducibility and construct validity of the Brazilian Portuguese version of the ATRS. Test-retest reproducibility was performed by assessing each participant on two separate occasions. The construct validity was determined by the correlation index between the ATRS and the Orthopedic American Foot and Ankle Society (AOFAS) questionnaires.

**Results:**

The final version of the Brazilian Portuguese ATRS had the same number of questions as the original ATRS. For the reliability analysis, an ICC_(2,1)_ of 0.93 (95 % CI: 0.88 to 0.96) with SEM of 1.56 points and MDC of 4.32 was observed, indicating excellent reliability. The construct validity showed excellent correlation with *R* = 0.76 (95 % CI: 0.52 to 0.89, *P* < 0.001).

**Conclusion:**

The ATRS was successfully cross-culturally validated into Brazilian Portuguese. This version was a reliable and valid measure of function in patients who suffered complete rupture of the Achilles Tendon.

**Electronic supplementary material:**

The online version of this article (doi:10.1186/s13102-016-0034-0) contains supplementary material, which is available to authorized users.

## Background

The use of patient-reported questionnaires to estimate functional capacity, pain and limitation in activities of daily living is an important outcome measure to monitor treatment progress in clinical and research settings [1]. The Knee Injury and Osteoarthritis Outcome Score (KOOS) [2] and the Foot and Ankle Outcome Score (FAOS) [3] are examples of patient-reported questionnaires widely used to assess perceived activity limitations in patients with osteoarthritis of the knee and patients with ankle ligament injury respectively. With increasing number of publications from different countries, cross-cultural adaptation of patient-reported questionnaires is essential when the aim is to compare the results of interventions across different populations [4, 5].

Ruptures of the Achilles tendon are relatively common in adults, especially in individuals aged 30–50 years. Men are three to four times more likely to sustain an injury than women [6–8]. The incidence has been reported to vary between 9.9 to 37.3 cases per 100,000 people [6, 9]. The recent increase in incidence has been associated with increased participation in high demand competitive and recreational sports, aging population, lack of fitness and performing strenuous physical activities [10–12]. Another explanation may be the increasing incidence of metabolic and other chronic diseases, which are associated with acute Achilles tendon ruptures [13].

An important tool for evaluation of patients who have suffered a complete rupture of the Achilles tendon is the Achilles Tendon Total Rupture Score (ATRS). The ATRS is a valid questionnaire with high reproducibility and sensitivity for measuring outcome after treatment in patients with total Achilles tendon rupture [14]. The ATRS was originally developed in Swedish, and has been translated and cross-culturally adapted into several languages, including Danish [15], and British English [16, 17].

To facilitate comparison among studies at an international level, cross-cultural adaptation of this questionnaire for the Brazilian population is warranted. The aim of this study was to perform the translation and cross-cultural adaptation of the Achilles Tendon Total Rupture Score (ATRS) into Brazilian Portuguese, and to determine its reproducibility and validity.

## Methods

### Design

This is a cross sectional study approved by the Ethics and Research Committee of our institution (Hospital Mater Dei, Belo Horizonte, Minas Gerais, Brazil). Patients from three tertiary hospitals were included into this study. All were examined for the purposes of the present study in our centre (Orthopedic Department, Hospital Mater Dei, Belo Horizonte, Minas Gerais, Brazil). All patients gave written informed consent to participate in this study.

### Translation

The ATRS is a self-reported questionnaire composed of ten items that reflect the opinion of patients regarding their symptoms, limitations in daily activities and physical activities after a complete rupture of the Achilles tendon. At the end of each question, the patient is given a scale from 0 to 10, where 0 corresponds to more symptoms and greater limitations of physical activity, and 10 indicates no symptoms and limitations. The final score is obtained by the sum of the responses for each item. The ATRS total score ranges from 0 to 100, with higher scores equal to less symptoms and limitations [10] (Additional file 1).

**Fig. 1:**
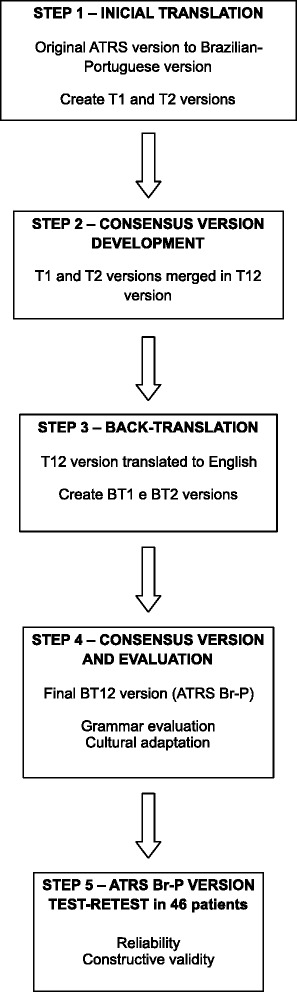
Five-step approach used to translate the Achilles Tendon Total Rupture Score (ATRS)

The cross-cultural adaptation of the ATRS was divided into 5 phases: initial translation (two bilingual Brazilian translators), synthesis of translation, back-translation (two native English language translators), consensus version and evaluation (expert committee), and testing phase [4] (Fig. 1). The first phase was the initial translation of the ATRS into the Brazilian-Portuguese, by two Brazilian bilingual translators, who produced two distinct versions, T1 version and T2. Only one of the translators had prior knowledge of the content of questionnaire [18].

The second stage was the synthesis of the translation, where the T1 and T2 versions were discussed with both translators, thus producing a single translation, the T1-2 version.

In the third phase, two bilingual translators, whose native language was English, translated separately the T1-2 version back into English, which results in the BT1 and BT2 versions. These two translators had no previous contact with the original questionnaire. This new translation into English allowed us to identify possible translation errors and grammatical inconsistencies, and was compared with the original version [19, 20].

The fourth phase consisted of the evaluation of all reports by an expert committee, which was composed by the authors of this work. The purpose of this phase was to review all translations to obtain a single version. For that purpose, the semantic equivalence, with regard to the meanings of words, with attention to idioms and colloquialisms, experimental equivalence, meaning to match activities of daily living in different countries and cultures and conceptual equivalence, ensuring the words have the same definition, were evaluated.

The final phase was the testing phase, where the final version of the Brazilian-Portuguese version of the ATRS was administered to 46 patients who suffered a total rupture of the Achilles tendon between the years 2007 and 2012 in 3 private hospitals in MG, Brazil. After answering the questionnaire, patients were asked about the difficulty of interpreting the questions or any other limitation in understanding the questionnaire.

### Test-retest reproducibility

This test-retest reproducibility of the Brazilian Portuguese version of the ATRS was conducted with a minimum interval of one week and a maximum interval of two months for each patient. The coefficient of Intra-Class Correlation (ICC) type 2,1 was used to determine the reproducibility of ATRS. The strength of agreement was classified using the following benchmarks: poor reproducibility for values below 0.40, good reproducibility for values between 0.40 and 0.75, and excellent reproducibility for those values above 0.75 [21]. We also calculated the standard error of the measurement (SEM) and the minimal detectable change (MDC) as additional measures. The SEM and MDC were calculated as follows: SEM = s√1 − ICC (where s is the standard deviation of the baseline) [21] and MDC = 1.96 × √2 × SEM, respectively [14, 22]. The SEM reflects the error of the instrument; and the MDC reflects the smallest within-person change in a score that, with *p* < 0.05, can be interpreted as a “real” change, above the measurement error.

### Validity

The construct validity, which is the ability of the instrument to measure an abstract concept, [21] in this case, functional limitation, was determined by the Pearson coefficient between the ATRS and Orthopedic American Foot and Ankle Society (AOFAS) questionnaire for the hindfoot. The AOFAS questionnaire, already translated into Portuguese, was developed to evaluate the different anatomic regions of the foot, including the ankle and hindfoot. The questionnaire consists of nine items, divided into three categories: pain (40 points), functional aspects (50 points) and alignment (10 points), totaling 100 points [23]. In the analysis of construct validity, the Pearson correlation coefficient was calculated and its 95 % confidence interval estimated using bootstrapping (1000 samples). Pearson correlation coeficient were interpreted as follow: values between 0.00 and 0.25 indicate little or no correlation, between 0.25 and 0.50 indicate low correlation, between 0.50 and 0.75 a moderate correlation, above 0.75 indicate excellent correlation [24].

To characterize the sample, the mean (range) for age, median (interquartile range) for time of assessment after surgery, and frequency for gender and limb affected were calculated. Mean and standard deviation as well as median and interquartile range were reported for ATRS data. Sample size calculation followed the proposed method by Walter et al. [25] for reliability studies. Based on an expected ICC of 0.85, two measures per participant on two separate days and accepting at least moderate reliability coefficient (ICC = 0.7), a total of 46 participants was required. Wilcoxon paired test was used to compare test-retest data since the score presents in ordinal data. Independent *t* test and Chi square test were used to compare groups for age, gender and ATRS variables, respectively. IBM SPSS (IBM Corporation, Somers, NY) software 18.0 version was used for all statistical analyses.

## Results

Forty-six patients with complete rupture of the Achilles Tendon were recruited. This group of patients was composed of 39 men (84.7 %) and 7 women (15.3 %), with the left lower limb being slightly more frequently affected (52 %). The average age of participants was 49 (range: 26–63) years. The median (interquatile range) time of assessment was 32.5 (26) months after the surgery.

During the process of translation and cultural adaptation of the ATRS, none of the patients assessed had doubts while answering the questionnaire, considering it self-explanatory, easy to understand and adapted to activities of daily living. No question was modified after the formal testing of the questionnaire, and the T1-2 version was considered the final version in Brazilian Portuguese of ATRS (Additional file 2).

From the total population sample, we analyzed data from 41 individuals undergoing test-retest after suffering total rupture of the Achilles Tendon. Five patients did not undergo the retest session, because we could not contact them or because they refused to answer the questionnaire again. For the reproducibility analysis, an ICC_(2,1)_ of 0.93 (95 % CI: 0.88 to 0.96) with SEM of 1.56 points and MDC of 4.32 was observed, indicating excellent reproducibility (Table 1).

**Table 1:** Test-retest reproducibility– Portuguese-Brazilian version of Achilles Tendon Total Rupture Score (ATRS)

ATRS (*n* = 41)	First session	Second session	Wilcoxon paired test (*p*-value)	ICC_(2,1)_ (95 % CI)
Mean (median)	94.3 (96)	93.8 (95)	0.2	0.93 (0.88 to 0.96)
SD (IQR)	5.9 (9.0)	6.1 (9.5)		

*SD* standard deviation, *IQR* interquartile range, *ICC* intraclasscorrelation coeficiente, *CI* confidence interval

**Fig. 2:**
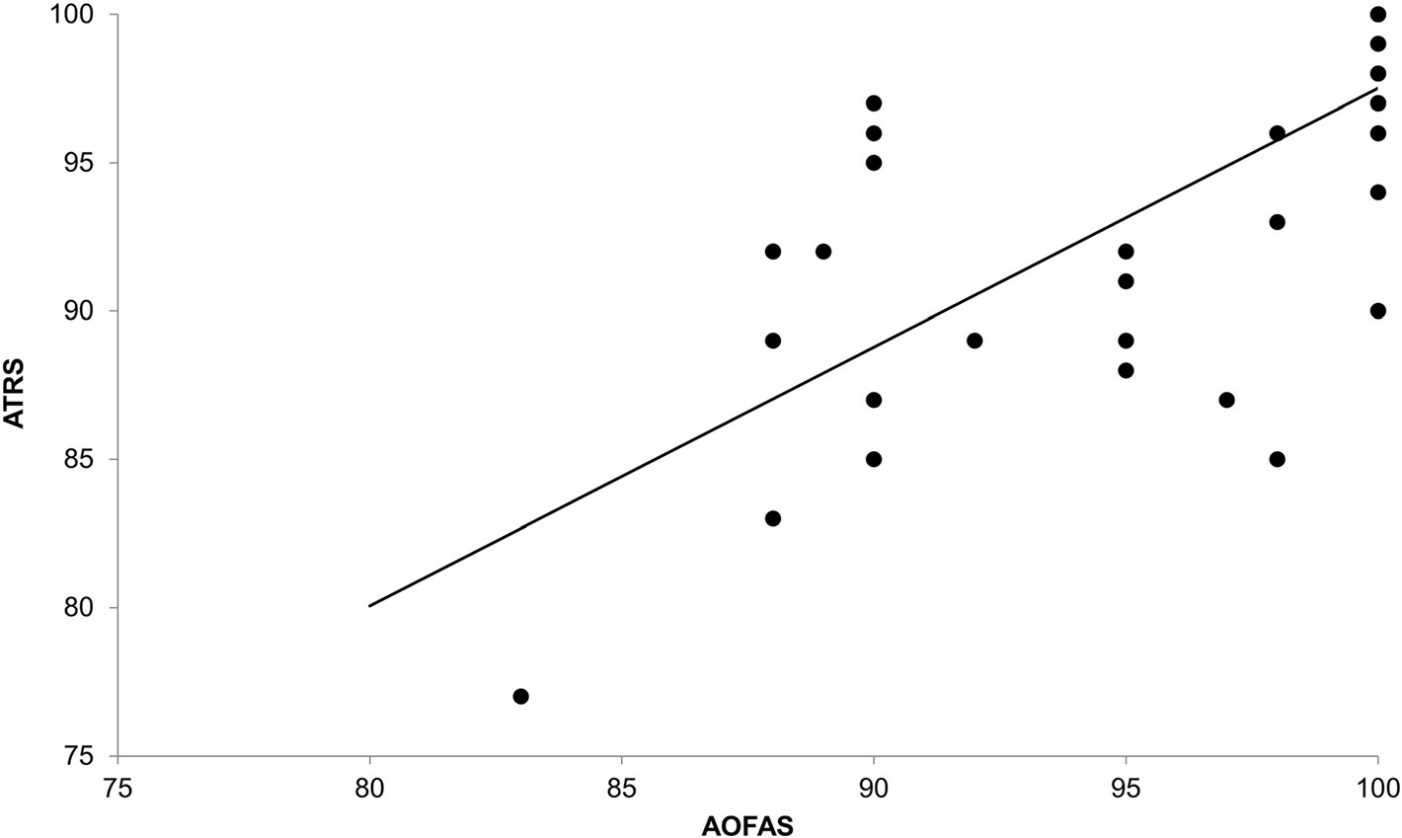
Correlation between Brazilian Portuguese version of Achilles Tendon Total Rupture Score (ATRS) and Brazilian Portuguese version of Orthopedic American Foot and Ankle Society (AOFAS)

Figure 2 shows the results for construct validity of the Brazilian-Portuguese version of the ATRS using the AOFAS score of 46 patients for the study as a reference. Using the Pearson correlation coefficient, we obtained an excellent correlation with *r* = 0.76 (95 % CI: 0.52 to 0.89), (*P* <0.001).

## Discussion

The most important finding of the present study is that, during the process of cross-cultural adaptation to Brazilian-Portuguese of the ATRS, no significant changes in any of the questions were needed. Patients were able to interpret all questions and understand the functional activities included in the questionnaire. The Brazilian-Portuguese version of the ATRS showed to be a reliable and valid tool to be used in the Brazilian population.

The reproducibility index found in this study is similar to the original version of the ATRS (ICC = 0.98), [10] the Danish version (ICC = 0.91), [15] and the British English version (ICC = 0.98) [16]. The MDC of 4.3 reported in this study was smaller than the one reported among the British English population [16] (MDC = 6.8), meaning that, to detect true change in the Brazilian population, clinicians should expect a difference of at least 4.3 between different time points.

The construct validity analysis showed an excellent correlation between the ATRS and the AOFAS with a Pearson coefficient of 0.76 (95 % CI: 0.52 to 0.89). Other questionnaires culturally adapted into Brazilian Portuguese have shown similar results for the analysis of construct validity. For instance, a study investigating the psychometric properties of the Brazilian Portuguese version of the Foot Health Status Questionnaire (FHSQ) found that five out of eight domains on the FHSQ were significantly correlated with Health Assessment Questionnaire and Numeric Rating Scale [19]. Similarly, the Brazilian Portuguese version of the American Orthopedic Foot and Ankle Society (AOFAS) Ankle-hindfoot scale showed moderate correlation with pain (*r* = 0.64, *p* < 0.01) and functional capacity (*r* = 0.67, *p* < 0.01) domains [23].

The present study has some limitations. Firstly, the time between the test and retest sessions was not the same for all patients. Patients were assessed with a minimum interval of one week or a maximum interval of two months. Some would argue that long intervals could have had a negative impact on the reproducibility index. However, the excellent reproducibility found in this study could be explained by the fact that the majority of patients were assessed at least one year after the surgery. At this point in time, patients might be less prone to change as improvements in functional level are expected, in most cases, in the early stages after surgery. Secondly, the only two psychometric properties investigated were reproducibility and construct validity. Future studies investigating other properties such as responsiveness and internal consistency are needed to study these properties in our Brazilian population.

## Conclusion

The cultural adaptation of the ATRS into Brazilian Portuguese showed that the new version is easy to apply, self-explanatory and with good ability to assess the functional limitations of patients who suffered a complete rupture of the Achilles tendon. This version will allow Brazilian clinicians to reliably compare the results of their patients with the international literature. Based on the results of the present study, we would encourage the use of the ATRS in both research and every day clinical work in a Brazilian Portuguese speaking population. Future studies are still needed in this area to determine whether the Brazilian-Portuguese version of the ATRS have similar responsiveness of other versions of the questionnaire.

### Ethics

The authors declare that the study received ethics approval. The raw data are available as Additional file 3.

## Data availability

The authors declare that the data are available, and the relevant data have been uploaded as Additional file 3.

## Additional files

Additional file 1: Appendix 1. Achilles Tendon Total Rupture Score (ATRS). (DOCX 482 kb) Additional file 2: Appendix 2. Achilles Tendon Total Rupture Score (ATRS). (DOCX 483 kb) Additional file 3: Raw data. (XLSX 16 kb)

## Abbreviations

AOFAS: Orthopedic American Foot and Ankle Society
ATRS: Achilles Tendon Total Rupture Score
FHSQ: foot health status questionnaire
ICC: Coefficient of Intra-Class Correlation
MDC: minimal detectable change
SEM: standard error of the measurement
