# Supplementary material for: Development of the Brazilian Portuguese version of the Achilles Tendon Total Rupture Score (ATRS BrP): a cross-cultural adaptation with reliability and construct validity evaluation
Source: BMC Sports Sci Med Rehabil. 2016 Apr 21;8:11. doi: 10.1186/s13102-016-0034-0 (PMC4839118; doi:10.1186/s13102-016-0034-0)
Supplement: Additional file 1: — Appendix 1. Achilles Tendon Total Rupture Score (ATRS). (DOCX 482 kb) [file 13102_2016_34_MOESM1_ESM.docx]

Appendix 1

Achilles Tendon Total Rupture Score (ATRS)

All questions refer to your limitations/difficulties related to your injured Achilles Tendon.

Mark with an X the value that corresponds to your level of limitation:

1. Are you limited due to decreased strength in the calf/Achilles tendon/foot?

0 1 2 3 4 5 6 7 8 9 10

2. Are you limited due to fatigue in the calf/Achilles tendon/foot?

0 1 2 3 4 5 6 7 8 9 10

3. Are you limited due to stiffness in the calf/Achilles tendon/foot?

0 1 2 3 4 5 6 7 8 9 10

4. Are you limited due to pain in the calf/Achilles tendon/foot?

0 1 2 3 4 5 6 7 8 9 10

5. Are you limited during activities of daily living?

0 1 2 3 4 5 6 7 8 9 10

6. Are you limited when walking on uneven surfaces?

0 1 2 3 4 5 6 7 8 9 10

7. Are you limited when walking quickly up the stairs or up a hill?

0 1 2 3 4 5 6 7 8 9 10

8. Are you limited during activities that include running?

0 1 2 3 4 5 6 7 8 9 10

9. Are you limited during activities that include jumping?

0 1 2 3 4 5 6 7 8 9 10

10. Are you limited in performing hard physical labor?

0 1 2 3 4 5 6 7 8 9 10
