# Supplementary material for: Development of the Brazilian Portuguese version of the Achilles Tendon Total Rupture Score (ATRS BrP): a cross-cultural adaptation with reliability and construct validity evaluation
Source: BMC Sports Sci Med Rehabil. 2016 Apr 21;8:11. doi: 10.1186/s13102-016-0034-0 (PMC4839118; doi:10.1186/s13102-016-0034-0)
Supplement: Additional file 2: — Appendix 2. Achilles Tendon Total Rupture Score (ATRS). (DOCX 483 kb) [file 13102_2016_34_MOESM2_ESM.docx]

Appendix 2

Achilles Tendon Total Rupture Score (ATRS-Br)

Todas as questões referem às suas limitações / dificuldades em relação ao Tendão de Aquiles lesado.

Marque com um X o valor que corresponde ao seu nível de limitação:

1. Você se sente limitado devido diminuição de força na panturrilha/tendão de Aquiles/pé?

0 1 2 3 4 5 6 7 8 9 10

1. Você se sente limitado devido a fadiga na panturrilha/tendão de Aquiles/pé?

0 1 2 3 4 5 6 7 8 9 10

1. Você se sente limitado devido a rigidez da panturrilha/tendão de Aquiles/pé?

0 1 2 3 4 5 6 7 8 9 10

1. Você se sente limitado devido a dor na panturrilha/tendão de Aquiles/pé?

0 1 2 3 4 5 6 7 8 9 10

1. Você se sente limitado durante as atividades cotidianas?

0 1 2 3 4 5 6 7 8 9 10

1. Você se sente limitado quando anda em superfícies irregulares?

0 1 2 3 4 5 6 7 8 9 10

1. Você se sente limitado quando caminha rapidamente, subindo escadas ou morro?

0 1 2 3 4 5 6 7 8 9 10

1. Você se sente limitado durante atividades que incluem corrida?

0 1 2 3 4 5 6 7 8 9 10

1. Você se sente limitado durante atividades que incluem saltos?

0 1 2 3 4 5 6 7 8 9 10

1. Você se sente limitado quando realiza trabalho físico pesado?

0 1 2 3 4 5 6 7 8 9 10
